# Analysis of Functional Single-Nucleotide Polymorphisms (SNPs) and Leaf Quality in Tea Collection under Nitrogen-Deficient Conditions

**DOI:** 10.3390/ijms241914538

**Published:** 2023-09-26

**Authors:** Lidiia Samarina, Jaroslava Fedorina, Daria Kuzmina, Lyudmila Malyukova, Karina Manakhova, Tatyana Kovalenko, Alexandra Matskiv, Enhua Xia, Wei Tong, Zhaoliang Zhang, Alexey Ryndin, Yuriy L. Orlov, Elena K. Khlestkina

**Affiliations:** 1 Center of Genetics and Life Sciences Sirius University of Science and Technology, Olimpiyskiy Ave. b.1, 354340 Sirius, Russia; q11111w2006@yandex.ru (L.S.); fedorinajv@talantiuspeh.ru (J.F.); kuzminad16@gmail.com (D.K.); karina.khusniyarova@gmail.com (K.M.); ddfvkt@gmail.com (T.K.); director@vir.nw.ru (E.K.K.); 2 Federal Research Centre the Subtropical Scientific Centre of the Russian Academy of Sciences, 344002 Sochi, Russia; malukovals@mail.ru (L.M.); matskiv_a@mail.ru (A.M.);; 3 State Key Laboratory of Tea Plant Biology and Utilization, Anhui Agricultural University, Hefei 230036, China; xiaenhua@ahau.edu.cn (E.X.);; 4 Agrarian and Technological Institute, Peoples’ Friendship University of Russia, 117198 Moscow, Russia; 5 Federal Research Center N. I. Vavilov All-Russian Institute of Plant Genetic Resources (VIR), 196632 Saint Petersburg, Russia

**Keywords:** *Camellia sinensis*, candidate genes, caffeine, catechin, germplasm collection, SNP effect, variant calling, theanine, nitrogen uptake

## Abstract

This study discusses the genetic mutations that have a significant association with economically important traits that would benefit tea breeders. The purpose of this study was to analyze the leaf quality and SNPs in quality-related genes in the tea plant collection of 20 mutant genotypes growing without nitrogen fertilizers. Leaf N-content, catechins, L-theanine, and caffeine contents were analyzed in dry leaves via HPLC. Additionally, the photochemical yield, electron transport efficiency, and non-photochemical quenching were analyzed using PAM-fluorimetry. The next generation pooled amplicon–sequencing approach was used for SNPs-calling in 30 key genes related to N metabolism and leaf quality. The leaf N content varied significantly among genotypes (*p* ≤ 0.05) from 2.3 to 3.7% of dry mass. The caffeine content varied from 0.7 to 11.7 mg g^−1^, and the L-theanine content varied from 0.2 to 5.8 mg g^−1^ dry leaf mass. Significant positive correlations were detected between the nitrogen content and biochemical parameters such as theanine, caffeine, and most of the catechins. However, significant negative correlations were observed between the photosynthetic parameters (Y, ETR, Fv/Fm) and several biochemical compounds, including rutin, Quercetin-3-*O*-glucoside, Kaempferol-3-*O*-rutinoside, Kaempferol-3-*O*-glucoside, Theaflavin-3′-gallate, gallic acid. From our SNP-analysis, three SNPs in WRKY57 were detected in all genotypes with a low N content. Moreover, 29 SNPs with a high or moderate effect were specific for #316 (high N-content, high quality) or #507 (low N-content, low quality). The use of a linear regression model revealed 16 significant associations; theaflavin, L-theanine, and ECG were associated with several SNPs of the following genes: *ANSa*, *DFRa*, *GDH2*, *4CL*, *AlaAT1*, *MYB4*, *LHT1*, *F3*′*5*′*Hb*, *UFGTa*. Among them, seven SNPs of moderate effect led to changes in the amino acid contents in the final proteins of the following genes: *ANSa*, *GDH2*, *4Cl*, *F3*′*5*′*Hb*, *UFGTa*. These results will be useful for further evaluations of the important SNPs and will help to provide a better understanding of the mechanisms of nitrogen uptake efficiency in tree crops.

## 1. Introduction

Nitrogen (N) is an essential element that is a part of proteins, nucleic acids, enzymes, hormones, and bioactive compounds. Under insufficient N conditions, the synthesis of enzymes slows down, which leads to the disruption of biosynthesis, metabolism, and, as a result, decrease in yield [1,2]. It is well known that plants uptake nitrogen from soil as nitrates, ammonium, urea, or amino acids. The mechanisms of nitrogen assimilation and utilization are complex, especially in perennial tree crops. Moreover, different genotypes can assimilate nitrogen with different levels of efficiency. Under low nitrogen conditions, some varieties are characterized by better agricultural quality compared to others [1,3,4,5]. The identification of plant genotypes that are highly efficient with respect to nitrogen uptake and utilization is important for the sustainable agriculture [6,7]. 

Efficient nitrogen-utilizing plants have evolved adaptive mechanisms to detect nitrogen concentrations in soils [8,9,10] and systemic signals that come from other parts of plant, such as the distant roots or shoots [8,10,11,12,13,14,15,16,17]. Some transcription factors related to these processes have been identified, such as MADS-box TF *ANR1*, *AP2/ERF*, *TGA1/TGA4*, *TCP20*, *NLP7*,*MYB*, *LBD37/38/39*, *bHLH35*, *bHLH36*, *WRKY* [5,7,10,18,19]. In addition, various hormonal pathways are controlled by soil nitrate concentrations and, in turn, regulate morphological and physiological plant responses [20,21,22,23,24]. 

Tea plant (*Camellia sinensis* (L.) Kuntze) is an important agricultural tree crop that is widely grown in more than 52 countries and used to produce the world’s second most popular non-alcoholic beverage [25,26,27]. Over the last 20 years, the world’s tea processing has doubled with the growth of the world’s population, and an increase in tea plantation demand has also taken place (from two to four million hectares) [28]. Tea quality depends on the contents of bioactive compounds such as polyphenols, caffeine, and theanine, amino acids, volatile compounds, and alkaloids [4,29,30,31,32,33,34,35], which underly the delicious taste, pleasant flavor, and beneficial health effects of tea [36,37,38,39,40,41,42,43]. Transcriptome research has provided new insights into the metabolic pathways and key genes involved in the biosynthesis, transport, and metabolism of catechins, theanine, and caffeine in tea plant tissues, as well as their relationship with genes that regulate abiotic stress responses [44,45,46,47,48,49,50,51,52]. 

On the one hand, the further expansion of tea farming is limited due to the lack of appropriate plantation areas with sufficient nitrogen supply. On the other hand, the excessive use of nitrogen fertilizers in tea plantations can cause damage to the environment [53], leading to soil acidification [54] and the accumulation of high levels of aluminum, fluorine, and heavy metals (e.g., chromium) in tea leaves, posing potential risks to human health [55]. Additionally, the long-term application of large amounts of N fertilizers can reduce soil fungal diversity in tea plantations [56]. Thus, the identification of nitrogen-efficient tea genotypes is critically important to maintain quality and yield under nitrogen-deficient conditions. Such genotypes can help to reduce the need for nitrogen fertilizers and expand the potential of tea production. In this context, it is necessary to analyze the differences within tea plant germplasms, which can affect nitrogen uptake efficiency and utilization [6]. 

The current knowledge of the relationships between the elements and metabolites in tree crops, particularly in tea, is still fragmentary. Recently, a set of genes involved in nitrogen metabolism in plants, including tea, were identified. Specifically, the co-expression of key genes such as *AMT*, *AQP*, *NRT*, *GOGAT*, and *GS* regulates ammonium uptake and assimilation [4]. Moreover, N-transporter genes, along with *GS* and *GOGAT*, are responsible for the regulation of theanine and glutamine contents [4]. Through employing the Kyoto Encyclopedia of Genes and Genomes (KEGG) analysis, the authors of [6] revealed that differentially expressed genes (DEGs) are primarily related to “nitrogen metabolism”, “amino acid metabolism”, and “hormone signal transduction”. For tea plants, the high-affinity uptake system is considered to be the main control factor for tea plant N metabolism. The regulators of soil N uptake (*AMT1.2*, *NRT2.4*, and *PIP)* and the regulators of N assimilation into amino acids in leaves and young shoots (*GS1.2*, *GDHa*, *GDH2*) and root-amino-acid transporters (*LHT1*) have been identified [5,6,33,57,58,59]. Additionally, amino-acid transporters such as *AAPs*, *LHTs*, *CATs*, *ProTs*, and *UMAMITs* play important roles in the N assimilation [60]. However, the polymorphisms in these genes have not been evaluated in genotypes with different N efficiencies. The goal of this study was to analyze the diversity of and the SNPs in the key N metabolism genes and their correspondence with specific phenotypes in the collection of 20 tea genotypes in Northwest Caucasia.

## 2. Results

### 2.1. Phenotypic Evaluation of the Collection 

Among the 20 genotypes, significant differences were observed according to their leaf size, leaf N content, photosynthetic efficiency, and leaf quality. Fifteen genotypes were classified as extra large according to their leaf area (Table 1). These genotypes were separated into three groups: (a) those with a mean leaf area of 149 cm^2^ (tetra-ploidy genotype #619), (b) those with a mean leaf area of 83–112 cm^2^ (#1102, #316, #2697, Karatum, #1385, #212), and (c) those with a mean leaf area of 65–75 cm^2^ (#551, #157, #3180, #536, #1405, #1877, #582, #1467). Most of the genotypes in the (b) and (c) groups were characterized by an increased genome size and related to are tri- and aneuploidy (Supplementary Table S1).

**Figure 1:**
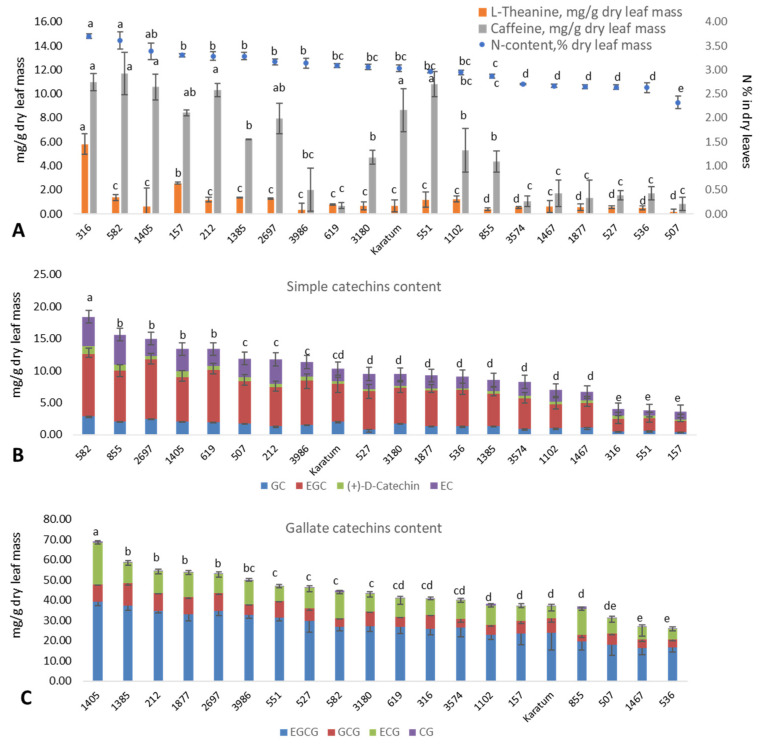
Leaf quality characteristics of the analyzed mutant forms. (**A**) Nitrogen content (%), theanine content (mg g^−1^), caffeine content (mg g^−1^) in dry leaf mass. (**B**) Simple catechins content (mg g^−1^ dry leaf mass). (**C**) Gallate catechins content (mg g^−1^ dry leaf mass). Different lowercase letters indicate significant differences according to Tukey’s range test (*p* ≤ 0.05).

Significant differences (*p* ≤ 0.05) in leaf N content were observed among the studied genotypes, with leaf N content values varying from 2.3 to 3.7%, and the mean standard deviation was 0.05–0.20% (Figure 1A). The highest leaf nitrogen content (3.4–3.7%) was detected in several mutant forms, namely #316, #582, and #1405. The lowest N-content (2.3%) was detected in the #507 mutant form. The caffeine content (mg g^−1^ dry leaf mass) varied significantly among the genotypes, with values ranging from 0.7 to 11.7 mg g^−1^. The highest caffeine content (8.6–11.7 mg g^−1^) was observed in several genotypes with a high leaf nitrogen content, namely #316, #582, #1405, #212, #551, and cv Karatum. The content of L-theanine (mg g^−1^ dry leaf mass) varied significantly among the genotypes, with values ranging from 0.2 (in #507) to 5.8 (in #316) mg g^−1^. Additionally, most of the genotypes with a low N content (#3574, #1467, #1877, #527, #536, and #507) were also characterized by low caffeine and low theanine contents compared to the genotypes with greater N contents. Thus, 5.8 mg g^−1^ of L-theanine and 11.0 mg g^−1^ of caffeine were observed in #316. The lowest nitrogen (2.3%), theanine (0.19 mg g^−1^), and caffeine (0.84 mg g^−1^) contents were observed in the mutant form #507. However, the correspondence between the N content in the leaves and catechins were not clear. The highest contents of simple catechins (13–20 mg g^−1^) were observed in the following genotypes: #582, #619, #2697, #1405, and #855. The lowest content (about 5 mg g^−1^) was observed in #316, #551, and #157 (Figure 1B). The highest gallated catechin contents (55–70 mg g^−1^) were observed in #2697, #1405, #1385, #212, and #1877, and the lowest (25–35 mg g^−1^) were observed in #536, #1467, and #507 (Figure 1C). Thus, mutant forms #2697 and #1405 were exhibited the highest content of both simple and gallated catechins. 

**Figure 2:**
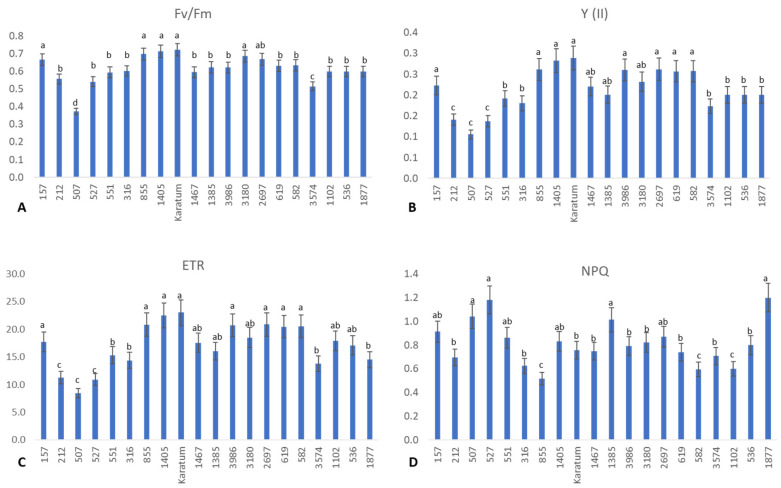
The efficiency of photosynthesis in 20 tea genotypes. (**A**) Photochemical yield (Fv/Fm). (**B**) Operating efficiency of the PSII. (**C**) Electron transport rate. (**D**) Non-photochemical quenching. Different lowercase letters indicate significant differences according to Tukey’s range test (*p* < 0.05).

Interestingly, significant differences were detected among the genotypes with respect to photosynthetic efficiency and the functional state of the PSII (Figure 2). The highest photochemical yield (about 0.7) was observed in Karatum, #157, #855, #1405, #3180, and #2697, and the lowest photochemical yields (about 0.4–0.5) were observed in #3574 and #507 (Figure 2A), indicating the differences in photosynthetic efficiency in these genotypes. Additionally, the highest operating efficiencies of PSII (Y) (about 20–30%) and the highest electron transport rates (ETRs) (about 16–23%) were detected in the following genotypes: #157, #855, #1405, cv Karatum, #1467, #3180, #3986, #2697, #619, and #582. On the other hand, the lowest Y (about 10%) and lowest ETRs (about 8–11%) were detected in three genotypes: #212, #507, and #527 (Figure 2B,C). Interestingly, the highest non-photochemical quenching (NPQ) value was detected in #507, #527, #1385, #157, #1405, #2697 and #1877, indicating a higher level of light energy to heat energy dissipation compared to the other genotypes. On the other hand, the lowest NPQ value was detected in #855, #582, and #1102, evidencing the higher level of photochemical energy utilization in these three genotypes compared to the other genotypes. 

**Figure 3:**
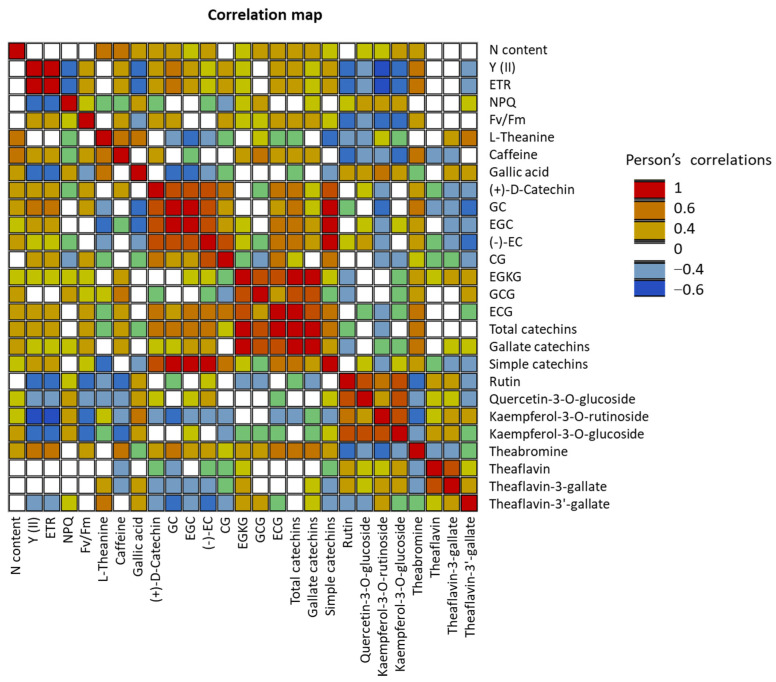
Pearson correlations between different biochemical parameters. Statistically significant Pearson correlations at *p* ≤ 0.05 are indicated by colored cells.

Significant positive correlations were detected between the nitrogen content and biochemical parameters such as L-theanine, caffeine, and most of the catechin contents (Figure 3). However, nitrogen content was not correlated with Y, ETR, NPQ, and Fv/Fm. Interestingly, significant negative correlations were detected between photosynthetic parameters (Y, ETR, Fv/Fm) and several biochemical compounds, such as rutin, Quercetin-3-*O*-glucoside, Kaempferol-3-*O*-rutinoside, Kaempferol-3-*O*-glucoside, Theaflavin-3′-gallate, and gallic acid.

### 2.2. Detection of SNPs in the Selected Genes and their Relationships with Phenotypes

A high level of variability in SNP number was observed in target genes (Table 2). The highest SNP density in the exone regions (about 2) was observed in three genes, namely *F3*′*5*′*Hb*, *4CL*, and *AMT1.2*, while the lowest SNP density (about 0.0–0.1) was detected in the exons of *bG*, *WD40*, *GDH2*, *LAR*, *AlaAT1*, *bHLH35*, *MYB7*, and *bHLH36*. Additionally, the highest rate of SNPS/exon (more than 30%) was detected in *F3*′*5*′*Hb*, *AMT1.2*, *DFRa*, *PIP*, and *bG*.

Interestingly, no polymorphisms were detected in *FLS*, *CHS*, and *ANRa*. 

**Figure 4:**
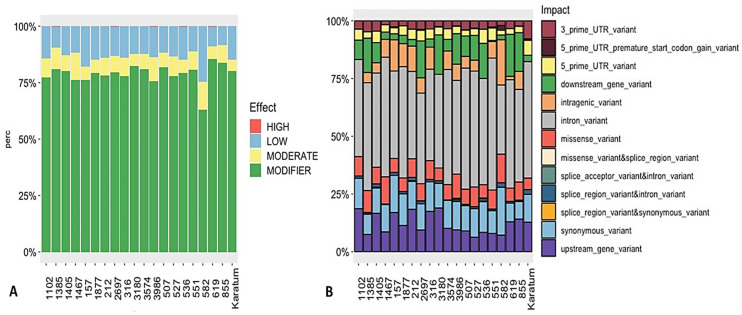
SNPs’ effect (**A**) and impact (**B**) on the phenotypes of 20 tea genotypes.

The SnpEff tool variation annotation was used to classify the SNPs according to their effect impact, and the SNPs were classified as either high-effect, moderate-effect, or low-effect or considered to have a modifier effect. In total, 0.2–0.3% of the SNPs were classified as a high-effect SNPs, and these were detected in the following genotypes: #619, #2697, #536, #1385, and #3986 (Figure 4A). Additionally, 7–12% were classified as moderate-effect SNPs, which were identified in all 20 genotypes. The majority of the SNPs were related to those with a modifier effect. An analysis of SNP distribution among the 20 genotypes indicated that 2–8% of the SNPs belonged to 3′-UTR (three prime untranslated region), and 2–6% belonged to the 5′-UTR variants (Figure 4B), with the highest percentage being observed in cv. Karatum. Additionally, broad distributions of the downstream (0.5–18.0%) and upstream (6.0–19.0%) gene variants were detected among the 20 genotypes. The highest percentages of the downstream gene variants (10–18%) were detected in #527, #3986, #1385, #536, #2697, #855, #3180, and #619, and the lowest percentages (0.5–3.0%) were detected in #551, #582, #157, cv Karatum, #212, #1467, #316, and #1877. The highest percentages of the upstream gene variants (11.0–19.0%) were detected in cv Karatum, #619, #855, #1405, #157, #316, #212, #1102, and #3180, and the lowest (6.0–8.0%) were detected in #527, #582, #1385, #551, and #536. In total, 0.2–2.1 of the detected SNPs were related to splice region variant and intron variants, with the highest percentages (2.0–2.1%) being observed in #1405, #582, and cv Karatum, and the lowest (0.2–0.3%) being observed in #3574, #3986, #536, and #212. Approximately 5.0–13.0% of the SNPs were detected as missense variants, with the highest levels (10.0–13.0%) being observed in #3986, #1467, #582, and #1385, and the lowest levels (5.0–6.0%) being observed in cv Karatum, #3180, #619, #536, #1877, and #157. Additionally, the lowest percentage of intragenic variants (1.5%) was detected in #619, and the highest percentages (10.0–20.0%) were observed in #212, #157, #582, #316, and #1877. Finally, 0.2–0.3% of the splice acceptor and intron variants were detected in the following genotypes: #3986, #619, #2697, #1385, and #536. 

**Figure 5:**
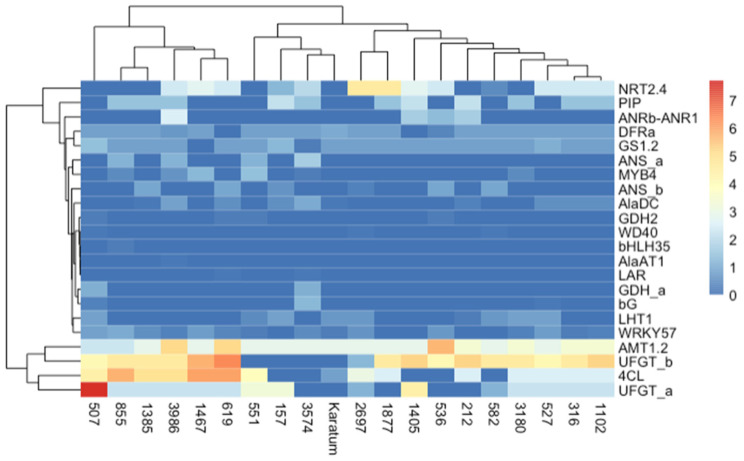
Heatmap of exon SNP frequencies calculated according to the following: SNP_freq = SNP_count_per_gene/gene length × 10^3^. The columns represent the tea genotypes, and the rows represent the different genes.

We generated heatmaps to compare the SNP frequencies in the exon regions of the studied genes (Figure 5). The heatmaps indicated that the highest frequencies occurred in *AMT1.2*, *UFGTa*, *UFGTb*, and *4Cl* in most of the genotypes, while the lowest frequencies were observed in *GDHa*, *GDH2*, *WD40*, *bHLH35*, *AlaAT1*, *LAR*, and *bG*. The application of the neighbor joining method to the studied mutant forms indicated two distinct branches, and one of them was divided into two sub-branches. The first branch consisted of six genotypes with the highest SNP frequencies, namely #507, #855, #1385, #3986, #1467, and #619. Most of them were characterized by a low N content and low caffeine and L-theanine contents. The second branch consisted of 14 genotypes and was divided into two sub-branches. Most of the genotypes with high leaf quality shared the same sub-branch. 

The sets of SNPs that were classified as high- or moderate-effect SNPs were finally identified (Table S3). Among them, two SNPs in *WRKY57* were most frequent among the most genotypes with a low nitrogen content and low tea quality. Moreover, 29 SNPs with a high or moderate effect were associated with #316 or #507 (two genotypes that contrast according to their leaf N content and leaf quality). Specifically, for #507, these specific SNPs were observed in the following genes: *GDHa* (1 SNP), *GDH2* (1), *WD40* (2), *4CL* (7), *F3*′*5*′*Hb* (3), *WRKY57* (2), and *UFGTa* (2). Additionally, a few specific SNPs were observed in #316, namely *4Cl* (1), *NRT2.4* (1), *F3*′*5*′*Ha* (1), *F3*′*5*′*Hb* (2), *AlaDC* (3), and *UFGTa* (1). Most of these SNPs may be responsible for changes to the biochemical properties of proteins. 

A linear regression model was applied to reveal the possible associations between the phenotypic data and the SNPs. This analysis led to the recording of 16 significant associations, and the level of significance was set at *p* value < 0.05 (Table 3). Theaflavin, L-theanine, and ECG were associated with several SNPs of the following genes: *ANSa*, *DFRa*, *GDH2*, *4CL*, *AlaAT1*, *MYB4*, *LHT1*, *F3*′*5*′*Hb*, and *UFGTa*. Among them, seven SNPs of moderate effect may be responsible for changes in the amino acid contents of the final proteins of the following genes: *ANSa*, *GDH2*, *4Cl*, *F3*′*5*′*Hb*, and *UFGTa*. 

## 3. Discussion 

The new regulatory approaches regarding plant mineral nutrition have been outlined to improve yield quality and quantity and are based on a desire to create cultivars that can effectively adapt to a specific level of soil fertility and are characterized by high nitrogen uptake and utilization efficiency [1,61]. Different plant genotypes can uptake and utilize soil nitrogen with different levels of efficiency [6,7]. Thus, the identification of these genotypes and the discovery of the mechanisms underlying high-level N deficiency are important to develop molecular markers and facilitate their further application in breeding programs [3,4,5].

In this study, twenty tea genotypes displayed significant differences according to their leaf size; ploidy level; and N, caffeine, L-theanine, and catechin contents. The genotype #507 was characterized by having the smallest leaf size; lowest N, L-theanine, and caffeine contents; lowest photochemical yield (Fv/Fm), operating efficiency of the PSII (Y), and Electron transport rate (ETR); and the highest non-photochemical quenching compared to the other genotypes. These results indicate this genotype’s low level of photochemical utilization of light energy, leading to the low photosynthetic capacity of this genotype [62,63,64]. The mutant form #316 was characterized by an extra-large leaf size and showed the highest N, caffeine, and theanine contents. This genotype was characterized by having average values of Y, ETR, and Fv/Fm compared to the other genotypes. The SNP distribution and frequencies of these two genotypes showed great dissimilarity, and the two were placed in two different branches, showing the highest genetic difference among these two genotypes.

Significant positive correlations were detected between the nitrogen content and biochemical parameters such as theanine, caffeine, and catechin contents. L-theanine and caffeine have been shown to be positively correlated with soil N content, which is consistent with our results [65]. However, according to our results, no negative correlation was observed between theanine and catechins. This is not consistent with the other studies in the literature, as some have reported that catechin and L-theanine contents are negatively correlated [66]. Surprisingly, in this study, we observed no correlations between nitrogen content and the parameters of photosynthetic efficiency (Y, ETR, NPQ, Fv/Fm).

The allele frequency data are useful for identifying the loci underlying phenotypic responses to selection or natural variation in phenotypes. The highest SNP frequencies were detected in the following genes: *UFGTa*, *UFGTb*, *4Cl*, and *AMT1.2*, indicating their high variability among tea accessions. Among them, the first three genes are related to tea quality; *UFGT* encodes UDP-flavonoid 3-*O*-glucosyl transferase and genes related to the anthocyanin biosynthesis pathway, while *4Cl* encodes 4-coumaric acid, CoA ligase, which participates in the biosynthesis of flavonoids [67]. *AMT1.2* is one of the key genes that encodes the ammonium transporter that regulates NH_4_^+^ uptake [68]. Tea plants have been reported to utilize ammonium more efficiently than nitrate, resulting in better growth [69,70]. However, nitrate-fertilized young shoots have been shown to exhibit a greater total catechin content and higher expression of genes encoding the flavonoid biosynthetic enzymes dihydroflavonol 4-reductase (DFR), chalcone synthase (CHS), and phenylalanine ammonia-lyase (PAL) compared to ammonium-fertilized shoots [71].

SNPeff can determine the different impact of SNPs [72]. A high-impact variant can cause function loss or gain, a premature stop codon, or a change in protein structure or function. A moderately significant variation may result in a non-disruptive change in protein function or structure. A low-impact variant may result in a silent mutation, which means that the genetic change has no influence on amino acid sequence or protein function. It could be a conservative missense variation with the same amino acid alteration. A modifier impact variant is one that is expected to alter the effect of another variant on the protein or to affect regulatory regions that control gene expression. Through analyzing SNPs with a high or moderate effect, we identified 18 SNPs that are unique to the low-quality genotype #507 in the following genes: *GDHa* (1 SNP), *GDH2* (1), *WD40* (2), *4CL* (7), *F3*′*5*′*Hb* (3), *WRKY57* (2), and *UFGTa* (2). Among them, only two SNPs in *GDH2* and *4CL* are significantly associated with theaflavin and ECG and promote changes in protein structures. As mentioned above, *4Cl* has been shown to participate in the flavonoid biosynthesis pathway, and this finding is consistent with our results. *GDH* encodes glutamate dehydrogenases, central enzymes in nitrogen metabolism, assimilating ammonia into glutamine or deaminating glutamate into α-oxoglutarate. Tea plant has two *GDH* genes: *CsGDH1* encodes the β-GDH subunit, and *CsGDH2/3* encode the α-GDH subunit, and their proteins all feature an NADH-specific motif [73].

To summarize, in this study, we revealed significant positive correlations between nitrogen content and biochemical parameters such as theanine, caffeine, and catechin contents. However, significant negative correlations between photosynthetic parameters (Y, ETR, Fv/Fm) and several biochemical compounds, such as rutin, Quercetin-3-*O*-glucoside, Kaempferol-3-*O*-rutinoside, Kaempferol-3-*O*-glucoside, Theaflavin-3’-gallate, and gallic acid, were observed. The application of a linear regression model revealed 16 significant associations; theaflavin, L-theanine, and ECG were associated with several SNPs of the following genes: *ANSa*, *DFRa*, *GDH2*, *4CL*, *AlaAT1*, *MYB4*, *LHT1*, *F3*′*5*′*Hb*, and *UFGTa*. Among them, seven SNPs of moderate effect led to changes in the amino acid contents of the final proteins of the following genes: *ANSa*, *GDH2*, *4Cl*, *F3*′*5*′*Hb*, and *UFGTa*. Among the 18 SNPs that were found to be unique to the low-quality genotype #507, only two SNPs (in *GDH2* and in *4CL*) were observed to have significant associations with theaflavin and ECG and promote changes in protein structure. Our results will be useful for further analyses of the associations of these SNPs in broad germplasm diversity with respect to tea collection and for the development of molecular markers for trait-oriented tea breeding.

## 4. Materials and Methods

### 4.1. Plant Materials and Phenotypic Evaluation

The plant materials were obtained from the field gene bank of the Federal Research Centre the Subtropical Scientific Centre of the Russian Academy of Sciences (FRC SSC RAS). Mutant forms derived in USSR between 1970 and 1980 via the γ-irradiation of seeds (mostly cv. “Kolkhida”, cv. “Qimen”) were included in this study (Table S1). All plants were about 31–33 years old. All plants were clonally propagated with 30–60 replicates per genotype and grown on brown forest acid soil (pH 5.5) with a nitrogen content of 30 mg kg^−1^ (compared to the optimal 80 mg kg^−1^ N for tea plantation). No fertilizers have been applied in the experimental plot for the last 27 years.

The leaf-related traits were characterized using the ten most fully expanded mature leaves collected from each cultivar and each replicate. The leaf area size was classified for all 106 genotypes of the entire tea collection according to Wang and Tang [74]: (1) small-leaf (leaf area ≤ 20 cm^2^); (2) middle-leaf (leaf area 20–40 cm^2^); (3) large leaf (leaf area 40–60 cm^2^); and (4) extra-large leaf (leaf area ≥ 60 cm^2^).

Photosynthetic efficiency was analyzed in the dark-acclimated leaves using the JuniorPAM chlorophyll-fluorometer with default settings. Ten mature leaves from each plant were included in the analysis. After applying actinic light, the following parameters were analyzed: Fv/Fm—maximum photochemical quantum yield of PS II; Y(II)—Effective photochemical quantum yield of PS II; NPQ—Stern-Volmer type non-photochemical fluorescence quenching; and ETR—electron transport rate [62,63,64].

The leaf nitrogen content in the mature leaves was analyzed spectrophotometrically using the Kjeldahl method, which includes the digestion (samples were heated in the presence of sulfuric acid) and distillation of the solution and the conversion of ammonium salt to ammonia via the addition of sodium hydroxide, followed by trapping the distilled vapors in HCl–water solution. Finally, the amount of ammonia or the amount of nitrogen present in the sample was then determined via back titration via the neutralization of HCl using NaOH solution [75].

The contents of caffeine, L-theanine and catechins (gallocatechin (GC), epigallocatechin (EGC), epicatechin (EC), epicatechin gallate (ECG), gallocatechin gallate (GCG), and epigallocatechin gallate (EGCG)) (mg g^−1^ dry leaf mass) were evaluated via HPLC using the following extraction protocol: adult tea leaves (3–4 leaves from the top of the branch) were fixed via steam treatment at 100 °C for 20 min in a water bath and subsequently dried. Approximately 200 mg of dried tea leaves were placed into a hermetically sealed container containing 4.0 mL 80% methanol-water solution, which was subsequently incubated for one week at +4 °C in the dark. After that, the vessels with the methanol leaf extracts were placed in a UV bath for 30 min and then centrifuged at 13,000× *g* rpm 10 min. A total of 1 mL of supernatants were injected into a HPLC column. The Agilent Technologies 1100 HPLC chromatographer, equipped with a flow-through vacuum degasser G1379A, 4th channel low-pressure gradient channel pump G13111A, automatic injector G1313A, column thermostat G13116A, and diode array detector G1316A, was used. The 2.1 × 150 mm column filled with octadecyl silyl sorbent, grain size of 3.5 µm, “ZORBAX-XDB C18” was applied. The acetonitrile solution was used for the gradient; the initial composition of the mobile phase, consisting of 90% (*v*/*v*) of solvent A (0.1% H_3_PO4) and 10% of solvent B (90.0% acetonitrile), was maintained for 8 min. Solvent A was then decreased linearly to 40% at 25 min and 0% at 90 min before being increased to 100% at 29.1 min to 34 min. Programming was then continued in the isocratic mode as follows: 40% A at 70.1 to 75.0 min and 7% A at 75.1 to 90.1 min (flow rate of 0.30 mL/min, column temperature of 40 °C). The detection wavelengths were 195 nm for L-theanine and 273 nm for caffeine and catechins. The identification of the substances was performed based on the holding time of the standards of the respective compounds.

### 4.2. Gene Selection and Primer Design and Long-Range Polymerase Chain Reaction (LR-PCR)

Thirty target genes were selected from the literature data (Table S2). The flanking primers were designed based on the reference tea genome *Camellia sinensis* var. *sinensis* cv. Shuchazao [76,77] using the following instruments: Primer3web https://primer3.ut.ee/ (accessed on 21 September 2023), GeneAlign https://pubmed.ncbi.nlm.nih.gov/16845010/ (accessed on 21 September 2023), OligoCalc https://pubmed.ncbi.nlm.nih.gov/17452344/ (accessed on 21 September 2023), https://www.ncbi.nlm.nih.gov/guide/sequence-analysis/ (accessed on 21 September 2023), https://molbiol-tools.ca/Alignments.htm (accessed on 21 September 2023), OligoAnalyzer https://eu.idtdna.com/pages/tools/oligoanalyzer (accessed on 21 September 2023), https://www.bioinformatics.org/sms/rev_comp.html (accessed on 21 September 2023) (Table 4).

The LR-PCR mixture of 20 µL consisted of 10 µL 2 × LR-PCR buffer containing a mix of HS-Taq and Pfu DNA-polymerases (Biolabmix, Novosibirsk, Russia https://biolabmix.ru/catalog/pcr/long-range/ (accessed on 21 September 2023), 0.3 µL (10 µM) of each primer (forward and reverse), sterile PCR water, and 1 µL of the DNA sample (50 ng µL^−1^). Amplification was performed in a MiniAmp thermal cycler (Thermo Fisher Scientific, USA) according to the following protocol: one cycle of preheating at 94 °C–4 min, 35 cycles of amplification (denaturation at 94 °C—20 s, annealing at 58–62 °C—20 s, elongation at 68 °C—2.5–5.5 min), and final elongation at 68 °C—10 min. The PCR products were separated in 2% agarose gel for 2.5 h at 90 V. After that, the fragments were cut out from the agarose gel, filtered through absorbent cotton [78], and then spined at 10,000× *g* for 15 min; 1/5 volume of acetate Na 3M and 80% volume of isopropanol were added, mixed, incubated vertically at −80 °C at 15 min, and centrifuged at 13,000× *g* for 20 min at +4 °C. Finally, the pellets were washed twice with 500 µL of 80% ethanol and dissolved in 10 µL of PCR water.

### 4.3. Pooled Amplicon Sequencing, Filtering and Variation Calling

For sequencing, one sample was obtained from each variety. To prepare fragment DNA libraries, we used PCR products that were obtained via the amplification of the target genes of tea collection. Fragment DNA libraries were prepared equimolarly from the mixed PCR products using the NEBNext Ultra II DNA Reagent Kit Library Prep Kit for Illumina according to the manufacturer’s protocol. Briefly, 526 ng of amplified PCR product was fragmented to 200–300 bp. using Covaris S220 with microTUBE-50 AFA Fiber Screw-Cap (Covaris, Woburn, MA, USA) in 50 µL of sterile water. Fragmented DNA was used for the further 3′ adenylation and ligation of the NEBNext Adapter for Illumina and following 3 cycles of amplification. A qualitative evaluation of the resulting libraries was carried out on an Agilent bioanalyzer TapeStation 4150 using High Sensitivity D5000 ScreenTape and High kits Sensitivity D5000 Reagents (Agilent, Santa Clara, CA, USA). A quantitative evaluation of the products was performed via real-time PCR using the KAPA Library Quantification Kit (KAPA biosystems, Wilmington, MA, USA).

The obtained fragments of the DNA library were mixed equimolarly into a pool and sequenced on the Illumina MiSeq via pair-end reads 76 + 76 bp. Sequencing data were demultiplexed via index sequences using the bcl2fastq v2.20.0.422 program with default parameters. In total, 184,000–392,000 pairs of reads were obtained for each DNA library. The initial quality assessment of the deep sequencing data was performed using the FastQC v0.11.2 software [79]. AdapterRemoval v2 programs [80] (with parameters --trimqualities, --minquality 20, --minlength 50) was used to remove adapters and low-quality sequences. A total of 94.34% of pairs of reads were preserved after filtering (Table S2).

Filtered data were mapped against the reference genome of tea plant (GCF_004153795.1). Mapping was performed using the bwa mem function from the package BWA programs [81]. The MarkDuplicates function of the picard-tools v2.22.2 (Picard toolkit [82]) software package was applied to remove duplicates. The quality of the alignments was evaluated using the Samtools v1.9 software package [83]. The depth coverage of the target genome regions was assessed using the CollectWgsMetrics function of picard-tools software package (https://broadinstitute.github.io/picard/ accessed on 21 September 2023) (with COVERAGE_CAP = 10,000 parameter). On average, 96.44% of reads were mapped to the tea genome. For each sample, on average, we obtained 229-fold coverage of the target genome regions of tea.

To control the raw read quality, FastQC (version 0.11.9) was used, and Trimmomatic (version 0.39) was employed with the parameters ‘ILLUMINACLIP:TruSeq3-PE-2.fa:2:30:10 LEADING:3 TRAILING:3 SLIDINGWINDOW:4:15 MINLEN:50’ to remove adapter sequences and low-quality sequences. The clean reads were aligned to the reference genome ‘Shuchazao’ with BWA-MEM (version 0.7.12) and sorted using SAMtools (version 1.16.1). To add read groups, GATK software (version 4.2) was used, and the GATK-HaplotypeCaller method was applied for variation calling. GATK software was used to select and filter SNPs/InDels according to the following parameters: ‘QD < 2.0||FS > 60.0||MQ < 40.0||SOR_filter||SOR > 4.0||DP < 10’ and ‘QD < 2.0||FS > 200.0||SOR > 10.0||DP < 10’, respectively. The database for the reference genome ‘Shuchazao’ was built using snpEFF (version 5.0) [72], which was then used to annotate the remaining variations. SnpEff tool variation annotation provided the effect impact classifications (high, moderate, low, or modifier). These impact variations are genetic variants predicted to cause a severe, moderate, low, or indirect effect on the protein.

SNP density was calculated as mean SNP per gene/fragment length of gene in kb. To obtain an overview of the SNP distribution and the possible enrichments of the SNPs for the genes, we normalized the SNP frequency in each gene. The SNP frequency in each gene was calculated using the following formula:

SNP_freq = (SNP_count/per_gene)/gene length × 10^3^

where SNP_count/per_gene is the amount of the SNPs detected in a certain gene, and gene_length is the length of the gene. The factor 10^3^ was applied to the denominator to leverage the SNP_Freq values in order to facilitate a fair and easy comparison.

### 4.4. Statistical Analysis

Statistical analyses of the data were carried out using XLSTAT software (free trial version) (https://www.xlstat.com/ Accessed on 21 September 2023). A one-way ANOVA, Student’s *t*-test, and Tukey’s test were applied to determine the significant differences between the variants. Additionally, hierarchical clustering was performed, and dissimilarities were calculated using the DICE coefficient (with agglomeration using Ward’s method). Additionally, a principal component analysis was conducted based on Pearson (n) correlations. To find the associations between SNPs and the phenotypes, a linear regression model was applied in conjunction with a statistical test adjusted for multiple comparisons (Bonferroni), with significant associations being noted at *p* values < 0.05.

## Figures and Tables

**Table 1:** Leaf trait characteristics of the analyzed mutant forms. Different lowercase letters indicate significant differences according to Tukey’s range test (*p* < 0.05).

Genotype	Breeding Status	Origin	Leaf Area, cm^2^ ± SD	Group
#507	mutant form	ɣ-mutant	40.8 ± 4.1 ^c^	Middle
#3574	mutant form	ɣ-mutant	46.1 ± 4.6 ^c^	Large
#855	mutant form	ɣ-mutant	55.6 ± 3.9 ^bc^	Large
#527	mutant form	ɣ-mutant	57.4 ± 5.0 ^bc^	Large
#3986	mutant form	ɣ-mutant	58.2 ± 5.1 ^bc^	Large
#551	mutant form	ɣ-mutant	65.6 ± 5.9 ^b^	Extra-large
#157	mutant form	ɣ-mutant	66.5 ± 5.8 ^b^	Extra-large
#3180	mutant form	ɣ-mutant	68.0 ± 6.0 ^b^	Extra-large
#536	mutant form	ɣ-mutant	68.7 ± 6.2 ^b^	Extra-large
#1405	mutant form	ɣ-mutant	69.8 ± 6.0 ^b^	Extra-large
#1877	mutant form	ɣ-mutant	70.6 ± 6.3 ^b^	Extra-large
#582	mutant form	ɣ-mutant	71.0 ± 6.3 ^b^	Extra-large
#1467	mutant form	ɣ-mutant	74.9 ± 6.7 ^b^	Extra-large
Karatum	Cultivar	Sport mutation	83.3 ± 6.5 ^ab^	Extra-large
#1385	mutant form	ɣ-mutant	83.8 ± 7.1 ^ab^	Extra-large
#212	breeding line	Clonal selection	84.6 ± 7.1 ^ab^	Extra-large
#1102	mutant form	ɣ-mutant	92.4 ± 8.3 ^a^	Extra-large
#316	mutant form	ɣ-mutant	97.3 ± 7.2 ^a^	Extra-large
#2697	mutant form	ɣ-mutant	112.3 ± 7.0 ^a^	Extra-large
#619	mutant form	ɣ-mutant	149.1 ± 7.3 ^a^	Extra-large

**Table 2:** The distribution of SNPs in the intron and exon regions of the target genes (N = 20). SNP density was calculated as mean SNP per gene/fragment length of gene in kb. Blue colors indicate lowest rates while red colors indicate highest rates of each parameter.

Gene	Fragment Length, bp	Mean SNPs Number in Introns	Mean SNPs Number in Exons	SNP Density in Introns	SNP Density in Exons	SNP % in Exons
*F3*′*5*′*Hb*	4435	6.25	7.60	1.41	1.71	54.87
*MYB4*	5342	8.85	01.05	1.66	0.20	10.61
*LHT1*	5107	3.50	1.40	0.69	0.27	28.57
*4CL*	5264	31.85	9.95	6.05	1.89	23.80
*AMT1.2*	2643	9.45	5.75	3.58	2.18	37.83
*GS1.2*	6202	58.05	4.85	9.36	0.78	7.71
*DFRa*	6600	4.45	4.35	0.67	0.66	49.43
*WRKY57*	11,214	41.85	4.25	3.73	0.38	9.22
*NRT2.4*	3060	10.50	4.15	3.43	1.36	28.33
*F3*′*5*′*H_a*	5118	11.35	3.25	2.22	0.64	22.26
*AlaDC*	7227	5.00	1.85	0.69	0.26	27.01
*PIP*	2006	2.25	1.25	1.12	0.62	35.71
*bG*	8605	0.20	0.65	0.02	0.08	76.47
*WD40*	3844	02.05	0.55	0.53	0.14	21.15
*GDH2*	4915	4.50	0.20	0.92	0.04	4.26
*LAR*	8600	0.45	0.1	0.05	0.01	18.18
*AlaAT1*	8058	1.85	0.05	0.23	0.01	2.63
*bHLH35*	5743	3.35	0.05	0.58	0.01	1.47
*MYB7*	3376	0.05	0.00	0.01	0.00	0.00
*bHLH36*	2953	0.25	0.00	0.08	0.00	0.00

**Table 3:** Significant associations between SNPs and the phenotypes at *p* value < 0.05. Italics indicate the SNPs with moderate effect where amino acids have changed. The SNPs specific for #507 are presented in bold italics.

SNPs	Gene	Position	Genotype (REF/ALT)	Phenotype	*p* Value	Amino (REF/ALT)	Property (REF/ALT)
SNP2	*ANSa*	1026570	G/T	L-Theanine	6.376794 × 10^−8^		
SNP3	*ANSa*	1026588	A/C	Theaflavin	3.458851 × 10^−6^		
*SNP6*	*ANSa*	1027478	A/G	Theaflavin	3.458851 × 10^−6^	p.Leu165Pro	Hydrophobic/ Unique
SNP11	*DFRa*	1286200	C/T	ECG/ Theabromine	0.0001012753/ 1.208252 × 10^−6^		
***SNP16***	*GDH2*	1343623	T/C	ECG/ Theabromine	0.0001012753/ 1.208252 × 10^−6^	p.Lys368Arg	Charged_basic/ Charged_basic
SNP67	*4CL*	2130618	C/A	L-Theanine	1.841082 × 10^−6^		
SNP184	*AlaAT1*	531832	A/G	L-Theanine	6.376794 × 10^−8^		
*SNP59*	*4CL*	2130419	A/G	Theaflavin	3.458851 × 10^−6^	p.Asn15Ser	Neutral/Neutral
***SNP60***	*4CL*	2130421	A/T	Theaflavin	3.458851 × 10^−6^	p.Thr16Ser	Neutral/Neutral
SNP68	*4CL*	2130888	T/C	Theaflavin	3.458851 × 10^−6^		
SNP120	*MYB4*	254145	A/G	Theaflavin	3.458851 × 10^−6^		
SNP187	*LHT1*	565216	G/A	Theaflavin	3.458851 × 10^−6^		
*SNP193*	*F3*′*5*′*Hb*	649286	T/A	Theaflavin	3.458851 × 10^−6^	p.Lys505Met	Charged_basic/ Hydrophobic
SNP194	*F3*′*5*′*Hb*	649465	G/A	Theaflavin	0.0001046889		
*SNP195*	*F3*′*5*′*Hb*	649575	G/A	Theaflavin	3.458851 × 10^−6^	p.Pro409Ser	Hydrophobic+ Unique/ Neutral
*SNP260*	*UFGTa*	883919	A/G	Theaflavin	3.458851 × 10^−6^	p.Thr274Ala	Neutral/ Hydrophobic

**Table 4:** Designed primers and LR-PCR conditions for the amplification of the selected genes.

Gene	Primer F 5′-3′	Primer R 5′-3′	Fragment Length, bp	Annealing Temp	Elongation, Min
*UFGT_a*	ACTCACAGGGATAAGAACACTC	CCATCTGGCAACATCTCCTC	1455	58	3
*UFGT_a*	ACTCACAGGGATAAGAACACTC	CACACTCCTCACCGCTCTT	1747	58	3
*PIP*	CCAAATGCCAAATATAGGGAGGC	GACCATAGAGAGAGTGGGTGG	2006	62	2.5
*CHS*	TTCGACCGCTTTGACCCGA	TGTTGCAGGCTCACATCACTC	2201	59	4
*UFGT_b*	TGATTGCTCATGTTATTTGGTCTC	TTGCCCATAACCTCTCCCTC	2600	62	3
*AMT1.2*	CCATTGCTGCTGAGACTGATAA	TGAGGTGATGGTGATTTGACGG	2643	61	2.5
*ANSa*	GGATGAAGGTGAGGGCAGAG	TGTCAGGGCTTTTTCTAAATCGT	2707	62	2.5
*bHLH36*	TCCATCAAAGAAGACAGACCACC	CCATTTGTCACCTTCTTGGTTTG	2953	60	3
*NRT2.4*	TTGTCCTTCCTTGGCTCCTTG	ACCAACTGTGCTCATTAACAAATC	3060	61	2.5
*MYB7*	GTTTGTGCTCTCACCGATTTCA	GTAGTAAACCCATACGTGGCCT	3376	59	3.5
*WD40*	AGGTCGGTGTAATTTCCGCT	ACAAGTGGCTAACTCTCATTGGT	3844	60	3
*ANS_b*	GTTGAAGGTTGCATTATTGAAGTTG	CCATGAGGTTTTGATTAGTGGAGT	3900	60	3
*F’3’5’Hb*	AGCCTCCTCTGTGGTAAGTG	CTCGACTCCACGAATACTAACA	4435	60	3.5
*GDH2*	GTCCTCCTATAACCTCCATTG	ATATTTGTTTTCTTGGTCACGTTTG	4915	58	3.5
*ANRb*	AGTCGTTGGTGCTGTGTGTT	AGGTTTACTTGGGTCTTCTTCAGG	5000	61	3
*ANRa*	GGTCCGTATGTGTGAATGAATCT	TGACTTGATCGTCGGAGTGC	5023	59	4
*LHT1*	GCTGTCTTCAGTAGCGGACTT	TTGCACGTTAGAATCAGGGTCAT	5107	58	5.5
*F3’5’Ha*	TGTGGTTGAGAAGAAAGGATACCA	GGAGACACCATAGCCGAAAGA	5118	58	3.5
*4CL*	GAGCTGCAATAGGCCTCACC	ACGACAGTAAAACCATAACAGAGT	5264	61	4
*MYB4*	TGACCCCTCATCACATTTAGT	CGTAAGTTCGTGGTGGTAGTG	5342	59	3.5
*FLS*	AGCAAGGCTATCGAAGGTGT	AGGGGAAGGTTTTAGGCAAG	5568	58	3.5
*bHLH35*	CGGAGAAATGATGGCGTGGT	GGCAAGTGGTTATGGGCGAA	5743	59	3.5
*GS1.2*	TGTTGGTTGATGTTGATGATGTT	CCATTTAAGTAGGAAGATTTCGCC	6202	61	4
*DFRa*	AATGTGGTGAAAGTGGACGTAG	TCTTGTTGCTGGATGACTGAC	6600	59	4
*AlaDC*	TGACCCACCCAAACAACGAG	TGGCACACACAAACGATAGTAGA	7227	59	6
*GDHa*	ACCATCCTAGTCACGCCCAT	CATGTATTTCCACAACAGAACAAG	7617	58	5.5
*AlaAT1*	GCTTCGTAAATGGAATCGCC	CCTTGGGCATTCGCAGCTT	8058	59	7
*LAR*	ACCTACACCAAAGACAACGAA	TGTTGTTATGGTGTTTGGTTGGTT	8600	58	6
*bG*	TCAGTAAGCCTTGAGAAAAGCA	GATGAAGTGTCACCTATTATGAGC	8605	59	6
*WRKY57*	TGATTCTTCTTGGGTCTAAACAG	GCCCACACTCACTCAAATCC	11,214	58	9

## Data Availability

All sequencing data are available in NCBI project # PRJNA97758.4.

## Supplementary Materials

The following supporting information can be downloaded at: https://www.mdpi.com/article/10.3390/ijms241914538/s1.
